# LOW INFECTION AND NON-UNION RATES IN POLYTRAUMA FEMORAL FRACTURES: A RETROSPECTIVE STUDY

**DOI:** 10.1590/1413-785220243202e278586

**Published:** 2024-06-24

**Authors:** Matheus Trindade Bruxelas de Freitas, Gabriel Benevides Valiate Martins, Matheus Augusto Maciel Santiago, Isaac Rocha Silva, Marcos de Camargo Leonhardt, Jorge dos Santos Silva, Kodi Edson Kojima

**Affiliations:** 1 Universidade de São Paulo, Faculdade de Medicina, Hospital das Clínicas (HC-FMUSP), Institute of Orthopedics and Traumatology, São Paulo, SP, Brazil.

**Keywords:** Multiple Trauma, Femoral Fractures, Intramedullary Nailing, Postoperative Complications, Traumatismo Múltiplo, Fraturas do Fêmur, Haste Intramedular, Complicações Pós-Operatórias

## Abstract

**Objective:**

Assess complications and risks in staged femoral shaft fracture treatment using external fixation and intramedullary nailing (DCO).

**Methods:**

Analysis involved 37 patients with 40 fractures, mostly male (87.5%), average age 32.9 years. Data included ASA score, AO/OTA and Gustilo classifications, Glasgow Coma Score, Injury Severity Score, times to external fixation and conversion, ICU duration, nail type, and reaming status. Complications tracked were mortality, deep infection, and non-union.

**Results:**

Predominant fracture type was AO/OTA A (45%), with 40% open (Gustilo A, 93.8%). Average ISS was 21; GCS was 12.7. Median ICU stay was 3 days; average time to conversion was 10.2 days. Retrograde nails were used in 50% of cases, with reaming in 67.5%. Complications included deep infections in 5% and non-union in 2.5%.

**Conclusion:**

DCO strategy resulted in low infection and non-union rates, associated with lower GCS and longer ICU stays. ***Level of Evidence III; Retrospective Cohort Study.***

## RESUMO

**Objetivo:**

Analisar taxa de complicações e riscos no tratamento estagiado de fraturas diafisárias do fêmur com fixador externo e conversão para haste intramedular (DCO).

**Métodos:**

Estudo com 37 pacientes, 35 masculinos, idade média de 32,9 anos, abordando escores ASA, classificação AO/OTA, Gustilo, Glasgow e ISS, tempo até a fixação externa, na UTI e tipo de haste. Complicações como mortalidade, infecção profunda e não união foram registradas.

**Resultados:**

Fraturas tipo AO/OTA A foram as mais comuns (45%), com 40% expostas (Gustilo A, 93,8%). ISS médio de 21 e ECG de 12,7. Média de 3 dias na UTI e 10,2 dias até a conversão. Uso de haste retrógrada em 50% dos casos e fresagem em 67,5%. As complicações incluíram infecção profunda em 5% e não união em 2,5%. A não união correlacionou-se com baixo ECG e tempo prolongado na UTI.

**Conclusão:**

A estratégia de DCO mostrou-se eficaz com baixas taxas de infecção e não união, associada a baixo ECG e tempo na UTI. ***Nível de Evidência III; Estudo de Coorte Retrospectivo.***

## INTRODUCTION

Polytraumatized patients often experience a systemic immunologic response due to their multiple injuries and associated hemorrhagic shock. When this response is not well-balanced, it can lead to acute complications, including respiratory distress syndrome and multiple organ failure.^1,2^ Among the factors that significantly impact the clinical course of these patients, major fractures, particularly femoral shaft fractures, stand out due to their potential for causing substantial bleeding and soft tissue damage.

The decision regarding how to stabilize femoral shaft fractures in polytraumatized patients is of paramount importance, as it can influence the final outcome. Early intramedullary nailing is the preferred approach for hemodynamically stable patients with good physiological reserves. In cases involving borderline hemodynamic stability or patients with limited physiological reserves, rapid stabilization using an external fixator, known as Damage Control Orthopedics (DCO), is an essential lifesaving measure that can also improve functional outcomes.^3,4^

However, the initial use of external fixation before definitive intramedullary nailing poses a potential risk of increased fracture complications, such as deep infections and non-union.

The objective of this retrospective study is to analyze the complication rate and identify the risk factors associated with femoral shaft fractures in polytraumatized patients who were initially treated with DCO (external fixation) and subsequently underwent intramedullary fixation. By investigating these complications and their contributing factors, we aim to provide valuable insights that can inform clinical decision-making and enhance patient care in this challenging population.

## CASUISTIC AND METHODS

This retrospective study has been performed at an urban university-based level one trauma center, between January 2019 and December 2021. Data were collected through a retrospective chart review and review of existing radiographs. Ethical approval was provided by the Scientific and Ethical Committee of the University under the protocol 12091. Written informed consent was obtained from all patients.

The inclusion criteria were as follows: age between 18 and 65 years, with femoral shaft fracture, Injury Severity Score (ISS) ≥ 16,^5^ submitted to damage control with external fixation, followed by definitive fixation with intramedullary nail, either closed or open Gustilo type I, II, IIIA and IIIB,^6^ minimum of 12 months of follow-up, and signed informed consent.

The exclusion criteria included pathologic fractures, proximal or distal femoral fractures, polytrauma without femoral shaft fracture, previous injury to the same limb, associated vascular injury, submitted to a different treatment protocol, open Gustilo type IIIC.

Demographic data on the following were collected: age, sex, body mass index (BMI), smoking habit, comorbidity, American Society of Anesthesiology score (ASA), fracture side, fracture classification according to the AO/OTA classification,^7^ location of the fracture in the shaft area, Gustilo classification for open fracture, Glasgow coma score (GCS), serum lactate, number of blood units transfused. Regarding the treatment the data collected were time to the external fixation, time to the definitive fixation, number of days in the intensive care unit (ICU), time with mechanical ventilation, respiratory complications (pneumonia, thromboembolism and acute respiratory distress syndrome), total days in the hospital, type of intramedullary nail, reamed or unreamed, post-operative infection, non-union and mortality.

Infection was defined according to the fracture-related infection criteria published by Metzemakers et al in 2018,^8^ and non-union was defined if the fracture was not healed within 6 months of follow-up. The qualitative parameters assessed were described for all patients using absolute and relative frequencies and the quantitative characteristics were described using summary measures (mean and standard deviation or median and quartiles). The occurrence of infection, non-union and poor outcomes (infection or non-union) were described according to the qualitative characteristics using absolute and relative frequencies and verified the association using Fisher's exact tests or likelihood ratio tests, the quantitative characteristics were described according to each outcome using summary measures and compared using Student's t-tests or Mann-Whitney tests according to the normality distribution of the data evaluated using Kolmogorov-Smirnov tests.^9^

The IBM-SPSS for Windows version 22.0 software was used to perform the analyzes and Microsoft Excel 2013 software was used to tabulate the data and make the graphs. The tests were performed with a significance level of 5%.

## RESULTS

During the observation period spanning from 2019 to 2021, a total of 37 patients presented with 40 femoral shaft fractures, all of which met the criterion of an Injury Severity Score (ISS) ≥ 16. These patients initially received damage control orthopedic treatment followed by definitive fixation with an intramedullary nail. Among the 37 patients included in this study, 35 (87.5%) were male, with an average age of 32.9 ± 9.4 years. Notably, three patients exhibited bilateral fractures, resulting in a total of 40 femoral shaft fractures. (Table 1)

**Table 1:** Demographic characteristics of the patients.

Variable	Description (n = 40)
Age (years)	
Mean ± SD	32.9 ± 9.4
Median (min.; max.)	32.5 (19; 60)
Gender, n (%)	
Female	5 (12.5)
Male	35 (87.5)
Body mass index (BMI) Kg/m^2^	
Mean ± SD	26.6 ± 4
Smoker	
n (%)	8 (20)
ASA score, n (%)	
I	24 (60)
II	14 (35)
III	2 (5)
Comorbidity	
n (%)	10 (25)
Fracture side, n (%)	
Right	22 (55)
Left	15 (37.5)
Bilateral	3 (7.5)
AO/OTA classification	
A	18 (45)
B	14 (35)
C	8 (20)
Gustilo classification, n (%)	
IIIA	15 (93.8)
IIIB	1 (6.3)
Injury Severity Score	
Median (range)	21 (16 - 50)
Glasgow Coma Score	
Mean ± SD	12.7 ± 4.1
Serum lactate (mmol/L)	
Mean ± SD	32.2 ± 20.2

The average Body Mass Index (BMI) among the participants was 26.6 ± 4 Kg/m², with only 8 individuals (20%) being smokers. The American Society of Anesthesiology (ASA) score distribution was as follows: ASA I in 24 patients (60%), ASA II in 14 patients (35%), and ASA III in 2 patients (5%). Ten patients (25%) had associated comorbidities. (Table 1)

Regarding the location of the fractures, 22 (55%) were on the right side, and 3 (8.1%) were bilateral. In accordance with the AO/OTA classification, 18 (45%) were classified as type A, 14 (35%) as type B, and 8 (20%) as type C. Among the 40 fractures, 16 (40%) were open; within this subset, 15 (93.8%) were categorized as Gustilo type A, and 1 (6.3%) as type B. The fractures were situated in the mid-portion of the shaft in 27 cases (67.5%), in the distal part of the shaft in 7 cases (17.5%), and in the distal shaft in 6 cases (15%). (Table 1)

The mean Injury Severity Score (ISS) was 21, ranging from 16 to 50. The average Glasgow Coma Score (GCS) was 12.7 ± 4.1, and the mean lactate level upon initial assessment in primary care was 32.2 ± 20.2 mmol/L. (Table 1)

The median duration of stay in the Intensive Care Unit (ICU) was 3 days, ranging from zero to seven days. Among the 40 patients, 13 (32.5%) required mechanical ventilation, with an average duration of 2.8 ± 2.9 days. Total hospitalization duration ranged from 9 to 58 days, with an average of 35.4 ± 29.9 days. Respiratory complications, including pneumonia, thromboembolism, and acute respiratory distress syndrome, were observed in 20 patients (50%). (Table 2) The average time interval between external fixation and intramedullary nailing was 10.2 ± 4.5 days, with a range of 3 to 24 days. All procedures were performed as one-stage interventions, involving the removal of the external fixator and subsequent fixation with an intramedullary nail. Among the 40 fractures, retrograde nails were utilized in 20 cases (50%), antegrade nails in 15 cases (37.5%), and cephalomedullary nails in 5 cases (12.5%). Reaming was performed in 27 fractures (67.5%). (Table 2)

**Table 2:** Results of the treatment.

Variable	Description (n = 40)
Time to external fixation (DCO), minutes	
Median (min.; max.)	30 (5; 54)
Time in the ICU, days	
Median (min.: max.)	3 (0; 7)
Mechanical ventilation, days	
n (%)	13 (32.5)
days (mean ± SD)	2.8 ± 2.9
Time in hospital, days	
Mean ± SD	35.4 ± 29.9
Time to definitive fixation, days	
Mean ± SD	10.2 ± 4.5
Type of nail, n (%)	
Retrograde	20 (50)
Antegrade	15 (37.5)
Cephalomedullary	5 (12.5)
Reaming, n (%)	
Reamed nail	27 (67.5%)
Deep infection	
n (%)	2 (5%)
Non-union	
n (%)	1 (2.5%)
Deep infection and non-union	
n (%)	1 (2.5%)

Deep infection was observed in two cases (5%), and non-union was identified in one case (2.5%) during the six-month follow-up. Notably, the latter case occurred in a patient who experienced paraplegia subsequent to a spinal cord injury. One fracture (2.5%) exhibited both deep infection and non-union. (Table 2)

Statistical analyses revealed that none of the patient or fracture characteristics exhibited a significant correlation with infection (p < 0.05). Furthermore, the time elapsed from external fixation to intramedullary nailing, although averaging 10 days, did not correlate significantly with the incidence of deep infection (p = 0.492).

Regarding non-union, statistical analyses indicated a correlation with a lower Glasgow Coma Score (p = 0.041) and an extended duration of stay in the ICU (p = 0.023). However, no significant correlations were observed between non-union and reaming (p = 0.242) or the type of nail employed (p = 0.452).

## DISCUSSION

Polytrauma constitutes a multifaceted and potentially life-threatening condition, necessitating a comprehensive and integrated approach. Traumatic injuries affecting the head, chest, abdomen, or pelvis often carry significant physiological repercussions. When coupled with a femoral shaft fracture, these cases become even more intricate due to soft tissue damage, hemorrhage, and the ensuing systemic inflammation, which elevate the risk of complications such as pulmonary infections, thromboembolic events, morbidity, and mortality.^10,11^ It is noteworthy that the presence of associated injuries escalates the 30-day mortality rate, reaching 12.8% when multiple injuries are present.^12^

In an effort to mitigate the exacerbation of the patient's systemic condition, damage control orthopedics (DCO) is employed as an effective strategy. DCO involves the initial application of external fixation for femoral shaft fractures, followed by definitive fixation once the patient's overall systemic condition, particularly the respiratory aspect, stabilizes. This approach has demonstrated its merit in reducing both morbidity and mortality.^13-16^

Our study corroborates the safety and efficacy of DCO in the management of polytraumatized patients with femoral shaft fractures. Importantly, none of the 37 patients in our cohort experienced mortality. However, it is crucial to recognize that while external fixation serves as an effective primary intervention, its continued application as the definitive treatment is not without risks and potential complications. These include loss of stability, mal-union, pin-track infections, and non-union.^17^ To mitigate these complications, the conversion to intramedullary fixation is recommended,^18,19^

A primary concern when transitioning to intramedullary nailing is the risk of infection. This risk is compounded by the presence of Schanz screws traversing soft tissues and breaching the cortex, thereby exposing the medullary canal to the external environment. Prolonged external fixation durations, in particular, elevate the risk of infection, as the trajectory of the Schanz screw may become susceptible to pin-track infection.^17^

Notably, while much literature emphasizes the life-saving benefits of DCO, there is a paucity of recent research focused on the long-term outcomes and complications in patients treated with this approach, especially in the context of femoral shaft fractures initially managed with external fixation and subsequently converted to intramedullary nailing.

In our study, three fractures (7.5%) developed deep infections, and notably, this did not correlate with several variables, including BMI, open fracture status, type of nail, reaming, or time to definitive fixation. Of significance is the lack of correlation between deep infection (Table 3) and the time to definitive fixation, which averaged 13 days in this subgroup.

**Table 3:** Deep infection statistical analyzes.

Variable	Deep infection		p
	No	Yes	
Age (years), mean ± SD	33.3 ± 9.7	28.8 ± 6.5	0.423
**Sex, n (%)**			
Female	4 (80)	1 (20)	0.338*
Male	33 (94.3)	2 (5.7)	
BMI (Kg/m^2^), mean ± SD	26.8 ± 4	24 ± 2.7	0.253
**Smoker, n (%)**			
No	29 (90.6)	3 (9.4)	> 0.999*
Yes	8 (100)	0 (0)	
**ASA, n (%)**			
I	23 (95.8)	1 (4.2)	0.469#
II	12 (85.7)	2 (14.3)	
III	2 (100)	0 (0)
**Respiratory complication**			
No	18 (90)	2 (10)	> 0.999*
Yes	19 (95)	1 (5)	
**Fracture side, n (%)**			
Right	21 (95.5)	1 (4.5)	0.498#
Left	13 (86.7)	2 (13.3)	
Bilateral	3 (100)	0 (0)	
**Comorbidities, n (%)**			
No	27 (90)	3 (10)	0.560*
Yes	10 (100)	0 (0)	
**AO/OTA classification, n (%)**			
A	17 (94.4)	1 (5.6)	0.349#
B	12 (85.7)	2 (14.3)	
C	8 (100)	0 (0)
**Gustilo classification, n (%)**			
IIIA	14 (93.3)	1 (6.7)	> 0.999*
IIIB	1 (100)	0 (0)	
**Glasgow coma score, n (%)**			**0.461** £
Serum lactate, median (min., max.)	29 (18; 43)	12 (6, 40)	0.136£
ISS, median (min., max.)	21 (17.5, 26.5)	22 (16, 28)	0.885£
**Fracture location in the shaft, n (%)**			
Proximal	5 (83.3)	1 (16.7)	0.439#
Median	25 (92.6)	2 (7.4)	
Distal	7 (100)	0 (0)
**Type of nail, n (%)**			**0.452** #
Retrograde	19 (95)	1 (5)	
Antegrade	13 (86.7)	2 (13.3)	
Cephalomedullary	5 (100)	0 (0)	
**Reaming, n (%)**			**0.242** *
No	11 (84.6)	2 (15.4)	
Yes	26 (96.3)	1 (3.7)	
Time to definitive fixation, days	10 (6, 13)	13 (8, 15)	0.492£
Time in the ICU, days median (min., max.)	3 (0, 6)	7 (0, 10)	0.626£
Time in the hospital, days median (min., max.)	24 (16, 37)	41 (23, 54)	0.251£

(£) Student t test,

(*) Mann-Whitney test,

(#) Fischer exact test,

Likelihood ratio test.

Comparatively, the rate of deep infection in closed femoral shaft fractures among non-polytrauma patients without a staged treatment approach has been reported as low in previous studies: 1% by Wolinsky et al.,^20^ 1% by Brumback et al,^21^ and 3% by Hammacher et al..^22^ Infection in nailing open femoral shaft fracture ranges from 2.4% and 4.8%.^23,24^ The staged treatment with conversion of external fixation to internal fixation with intramedullary nailing has historically shown higher infection rate: Taeger et al. 6.6%,^25^ Malik et al.,^26^ and Parekh et al 16%.^27^

Our findings further suggest that prolonged time for conversion may elevate the risk of infection, aligning with recommendations to keep the conversion period under two weeks.^28^

In our series, 2 fractures (5%) resulted in non-union, with correlations identified between non-union and lower GCS, longer ICU stays, and marginally with the time to conversion. These observations challenge the notion that cranial trauma promotes bone formation and subsequent healing. Importantly, we found no correlation between non-union and the type of nail or the reaming process. (Table 4) Our non-union rate is consistent with rates reported in previous studies: 3% shown by Nowotarski et al.,^29^ 6% by Malik et al.^26^ and 9% by Parekh et al.^27^

**Table 4:** Non-union statistical analyzes.

Variable	Non-union		p
	No	Yes	
Age (years), mean ± SD	32.4 ± 8.7	44.5 ± 21.9	0.577
**Sex,n (%)**			
Female	5 (100)	0 (0)	> 0.999*
Male	33 (94.3)	2 (5.7)	
BMI (Kg/m^2^), mean SD	26.5 4.1	27.5 1.2	0.743
**Smoker, n (%)**			
No	30 (93.8)	2 (6.3)	> 0.999*
Yes	8 (100)	0 (0)	
**ASA, n (%)**			
I	23 (95.8)	1 (4.2)	0.834#
II	12 (85.7)	2 (14.3)	
III	2 (100)	0 (0)
**Respiratory complication**			
No	19 (95)	1 (5)	> 0.999*
Yes	19 (95)	1 (5)	
**Fracture side, n (%)**			
Right	20 (90.9)	2 (9.1)	0.290#
Left	15 (100)	0 (0)	
Bilateral	3 (100)	0 (0)
**Comorbidities, n (%)**			
No	29 (96.7)	1 (3.3)	0.560*
Yes	9 (90)	1 (10)	
**AO/OTA classification, n (%)**			
A	18 (100)	0 (0)	0.266#
B	13 (92.9)	1 (7.1)	
C	7 (87.5)	1 (12.5)
**Gustilo classification, n (%)**			
IIIA	14 (93.3)	1 (6.7)	> 0.999*
IIIB	1 (100)	0 (0)	
Glasgow coma score, median (min., max.)	15 (3, 15)	6 (3, 9)	0.041£
Serum lactate, mean ± SD	29.5 ± 2	14.5 ± 2.5	0.085£
ISS, median (min., max.)	20.5 (16, 50)	25.5 (22,29)	0.885£
**Fracture location in the shaft, n (%)**			
Proximal	5 (83.3)	1 (16.7)	0.094#
Median	27 (100)	0 (0)	
Distal	6 (85.7)	1 (14.3)
**Type of nail, n (%)**			
Retrograde	19 (95)	1 (5)	0.744#
Antegrade	14 (93.3)	1 (6.7)	
Cephalomedullary	5 (100)	0 (0)
**Reaming, n (%)**			
No	12 (92.3)	1 (7.7)	>0.999*
Yes	26 (96.3)	1 (3.7)	
Time to definitive fixation, days	9.5 ± 4.5	34 ± 20	0.051£
Time in the ICU, days	3.0 ± 9.9	37.5 ± 17.5	0.021£
Time in the hospital, days	24.5 ± 25.4	80.5 ± 57.5	0.369£

(£) Student t test,

(*) Mann-Whitney test,

(#) Fischer exact test,

Likelihood ratio test.

Due the tight inclusion criteria of polytraumatized patient with ISS ≥ 16 with femoral shaft fracture treated initially with DCO the number of patients in our study was 37. Other studies also have shown a limited number of patients included: Nowotarski et al. 59 patients,^29^ Mallik et al, 12 patients,^26^ Taeger et al. 75 patients^25^ and Parekh et al. 16 patients.^27^

However, it is essential to acknowledge the inherent limitations in our study, including the relatively small sample size and the retrospective nature of data collection, which may introduce bias and imprecision. The absence of a control group for comparison further underscores the need for cautious interpretation of our results.

In conclusion, our study underscores the life-saving benefits of DCO in polytrauma patients with femoral shaft fractures. The conversion to internal fixation with intramedullary nailing emerges as a safe strategy, characterized by low infection and non-union rates. Nevertheless, the limitations inherent to our study, including its small sample size and retrospective design, necessitate the exercise of caution in interpreting and generalizing our findings. Further research, incorporating larger cohorts and prospective methodologies, is warranted to validate and refine our observations and treatment protocols.

## CONCLUSIONS

In our series the indication of damage control orthopedics in patients with femoral shaft fracture and ISS ≥ 16 lead to zero mortality. The conversion from the external fixation to the intramedullary nailing, done in average after 10 days, hasn't shown increase in infection and non-union rate. Non-union had correlation with lower GCS and longer stay in the ICU.
